# Clinical Study and Microbiological Analysis of Periodontopathogenic Microflora Analyzed among Children and Adolescents with Cardiovascular Diseases Compared to Group with Good General Status

**DOI:** 10.3390/pediatric16020041

**Published:** 2024-06-18

**Authors:** Oana Chipirliu, Marian Viorel Crăciun, Madalina Nicoleta Matei

**Affiliations:** 1 Research Centre in the Medical-Pharmaceutical Field, Faculty of Medicine and Pharmacy, Dunarea de Jos University of Galati, 47 Domneasca Str., 800181 Galati, Romania; 2 Research Centre in the Faculty of Automation, Computers, Electrical and Electronics Engineering, Dunarea de Jos University of Galati, 111 Domneasca Str., 800181 Galati, Romania

**Keywords:** subgingival microorganisms, real-time PCR, periodontal disease, cardiovascular diseases

## Abstract

Periodontal diseases, as an important part of oral pathology, present different characteristics when affecting children and adolescents or young adults. Studies have shown that adolescence and childhood are closely related to a high risk of periodontal disease, but the follow-up for periodontal health or damage at this age has been insufficiently appreciated until now. The aim of this study was to identify subgingival microorganisms using a real-time polymerase chain reaction (PCR) in a group of children and adolescents aged 7–17 years with and without cardiovascular disease. The group of 62 subjects with gingival inflammation and poor hygiene was divided into two groups according to general condition: 31 subjects with carduivascular disease (group A) and 31 subjects without cardiovascular disease (group C). Subjects were examined in the initial consultation, the state of hygiene and periodontal inflammation was assessed using the plaque index (PI) and gingival index (GI), and samples were taken from the gingival sulcus using sterile paper cones to determine nine subgingival microorganisms. Nine subgingival microorganisms were identified: *Aggregatibacter actinomycetemcomitans* (Aa), *Porphyromonas gingivalis* (Pg), *Treponema denticola* (Td), *Tannerella forsythias* (Tf), *Prevotella intermedia* (Pi), *Peptostreptococcus* (*Micromonas*) *micros* (Pm), *Fusobacterium nucleatum* (Fn), *Eubacterium nodatum* (En), and *Capnocytophaga gingivalis* (Cg). The patients were included in a specialist treatment program which aimed to relieve the inflammatory condition, remove local irritative factors, and train the patients to perform proper oral hygiene at home by using primary and secondary oral hygiene products. Subjects were reevaluated 3 months after treatment, when measurements for the PI and GI and microbiological determinations were repeated. The results showed a predominance of subjects aged 16–17 years (12.4%). Among the subjects with marked gingival inflammation, the male gender was predominant (58.06%). The PI values changed considerably after treatment, with lower values in patients presenting a general condition without cardiovascular disease (PI = 8.10%) compared with the patients with cardiovascular disease (PI = 13.77%). After treatment, the GI showed considerable changes in both groups. Red, orange, and purple complex microorganisms were found before treatment and decreased considerably after treatment in both groups. The highest values were found for *Treponema denticola* (140,000 (1.4 × 10^5^)) in patients with cardiovascular disease and generalized gingival inflammation. Of the pathogenic microorganisms, the most common was *Tannerella forsythia* in 52 patients before treatment, and red microorganisms considerably appeared in only 10 patients after treatment. *Capnocytophaga gingivalis* remained constant both in the diseased state and after treatment and was consistent with periodontal health. Children with cardiovascular diseases had a higher prevalence of gingival manifestations. The composition of the subgingival microbial plaque was directly influenced by the degree of oral hygiene, but the response to specialized treatment was also influenced by the general health status. The results of this study support the conclusion that periodontal pathogens appear and multiply in the absence of proper hygiene in childhood after the eruption of permanent teeth, and their action leads to the initiation of periodontal diseases.

## 1. Introduction

Oral biofilm is defined as a deposit adhering to dental and gingival surfaces made up of numerous microbial complexes, which are contained in an extracellular matrix [1,2,3,4]. The transition from periodontal health to periodontal disease is caused by the transformation of a highly organized subgingival bacterial community into a dysbiotic one [5,6].

The pathogenic potential of oral bacteria is not identical for all types of microorganisms or in all patients. In periodontally healthy individuals, periodontal pathogens colonize in low numbers and do not induce destructive lesions, being part of the normal oral microbial flora. The initiation of periodontal diseases occurs with the change in the proportion of subgingival bacteria and the pathological growth of periodontal pathogens [7,8]. Pathogenic bacteria that are involved in the initiation and worsening of periodontal disease are classified into three categories: (1) bacteria with high pathogenicity (*Aggregatibacter actinomycetemcomitans*, *Porphyromonas gingivalis*, *Treponema denticola*, and *Tannerella forsythia*); (2) bacteria with moderate pathogenicity (*Eikanella corrodens*, *Campylobacter rectus*, *Prevotella intermedia*, *Fusobacterium nucleatum*, and *Prevotella nigrescens*); and (3) bacteria with low pathogenicity (*Capnocytophaga ochracea*, *Capnocytophaga sputigena*, and *Capnocytophaga gingivalis*) [8,9,10,11,12].

Periodontal diseases, as an important part of oral pathology, present different characteristics when they affect children and adolescents or young adults [8,11,12,13,14]. The main types of periodontal disease encountered in childhood are specific forms of plaque-induced gingivitis, which are reversible when the plaque is removed [15,16,17,18,19]. The most prominent forms of gingival manifestations, resulting in bone damage and premature tooth loss, are often associated with chronic diseases and general disabilities, which have a major impact on the immune system [20,21,22]. Microbial plaque and the presence of periodontal pathogens are necessary for the initiation of periodontal disease, but host defense is an important factor coordinating the progression and severity of periodontal disease in individuals with systemic disease [22]. Socransky is the founder of the concept of bacteriology in the case of periodontal diseases, and the information introduced by him has undergone rapid development thanks to the new methods used for diagnosis, such as the polymerase chain reaction (PCR) or the genetic amplification reaction, for microbial identification specifically, and treatment and diagnosis of periodontal diseases have made great progress [8,23,24].

Through the PCR method, short fragments (of 100–2000 nucleotide base pairs) under the control of a DNA polymerase are subjected to several cycles of replication so that infinite enzyme amounts are amplified millions of times, becoming detectable with a DNA probe. Thus, even a single bacterial cell with a viral particle can be detected. The subgingival microorganisms studied by the DNA-AND chain hybridization method were grouped, depending on their virulence, into five complexes [8].

Few studies have focused on the bacterial species associated with the deterioration of dental and gingival health in children with congenital or acquired heart disease. Research has demonstrated a significant association between high periodontopathogenic microbial load, diagnosis of periodontal disease, and general health (FitzGerald in 2010, Nosrati et al. in 2013, and Mohamed Ali et al. in 2017) [25,26,27].

There are two important directions that follow the link between periodontal and cardiovascular diseases; the first follows the fact that bacteria and their toxins have a direct impact on the vessel wall during bacteremia, and the second assumes that cytokines and inflammatory mediators released during chronic periodontal inflammation can potentially negatively affect the vascular walls [28]. There is some evidence for alterations in the oral microflora as a result of physiopathological and treatment-related factors in children with congenital heart disease, but additional research is required to validate these findings [20,21,22,25]. The aim of this study was to detect the presence and quantify the nine periodontopathogenic bacterial strains using PCR assays. We performed the association of these microorganisms with local clinical parameters and the diagnosis of periodontal disease in a group of patients with cardiovascular disease compared to a control group of subjects without systemic pathology.

## 2. Materials and Methods

### 2.1. Study Populations

A total of 62 patients with gingival inflammation (GI greater than or equal to 1). Patients of both sexes aged 7–17 years and from urban areas were included in the study. Subjects were selected from patients of the children’s hospital in Galati and patients of a private dental practice in Galati. The subjects were divided according to the presence of a diagnosis of cardiovascular disease into subjects with gingival inflammation and cardiovascular disease (group A) and subjects with gingival inflammation and a good general condition (group C).

The study was carried out over a period of 18 months between May 2022 and November 2023. From the initial group consisting of 124 patients, for this study, only patients with various stages of gingival inflammation were selected.

What follows is our ethics statement. This study was designed and performed in compliance with the ethical principles for medical research involving human subjects in the Declaration of Helsinki. The research was approved by the relevant ethics committees. Informed consent was obtained, and each participant (legal representative) voluntarily signed the informed consent and clinical trial participation form. Patients were informed about the study in which they were invited to participate through an information form and detailed explanations about the research procedures and aims. The participants agreed to the necessary clinical and paraclinical investigations by signing an informed consent document to participate in this study.

### 2.2. Clinical Examinations

The periodontal status and dental hygiene status were recorded using the plaque index (PI) and gingival index (GI). Patients with PIs greater than or equal to 20% and GIs greater than or equal to 1 who had gingival inflammation of various stages were selected for this study. The specialist consultation was carried out with inspection, palpation, and percussion and included analysis of the superficial and deep marginal periodontium.

For clinical examination of the deep periodontium, a classic consultation kit (mirror, probe, tweezers, and spatula) and Williams-type buttoned periodontal probe were used, with the active part marked from in millimeters up to 3 mm, then 5 mm, 7 mm, and again in millimeters up to 10 mm. A periodontal survey was carried out to assess the following parameters: probing depth, the presence of bleeding on probing, gingival recession, and clinical level of attachment. For the age range of 7–12 years, the simplified periodontal screening was used, analyzing only six index teeth: the right maxillary central incisor, right and left maxillary first molars, left mandibular central incisor, and right and left mandibular first molars [29,30,31].

### 2.3. Sampling for PCR

Detection of the selected bacterial species involved collection of samples from the gingival sulcus and identification of periodontal pathogens. Harvesting was carried out cumulatively from five distinct points with the help of sterile paper cones from the harvesting kit, which was provided by the distributor MIP Pharma Romania. After completing the order form, the samples were sent to MIP Pharma GmbH (Muhlstrabe, St. Ingbert, Germany). The response with the results was received in approximately 10 working days after the samples were sent.

Harvesting was achieved by placing sterile paper cones (size: ISO 20) in the gingival sulcus and holding them there for approximately 10 s to absorb the pathological product from this level. The harvesting sites were dried and cleaned of supragingival plaque. The sampling was carried out in the areas that presented values greater than 2 mm during the periodontal survey. Supragingival plaque was removed using a sterile table or Gracey curette. The cones were stored and later transferred to Ependorf tubes. The team of the MIP Pharma laboratory, using the DNA-PCR technique, isolated seven microbial species with specific markers of the establishment of periodontal diseases—*Aggregatibacter actinomycetemcomitans* (Aa), *Porphyromonas gingivalis* (Pg), *Treponema denticola* (Td), *Tannerella forsythias* (Tf), *Prevotella intermedia* (Pi), *Peptostreptococcus* (*Micromonas*) *micros* (Pm), and *Fusobacterium nucleatum* (Fn)—and two bacterial species also compatible with periodontal health: *Eubacterium nodatum* (En) and *Capnocytophaga gingivalis* (Cg).

### 2.4. Extraction of DNA

**Figure 1:**
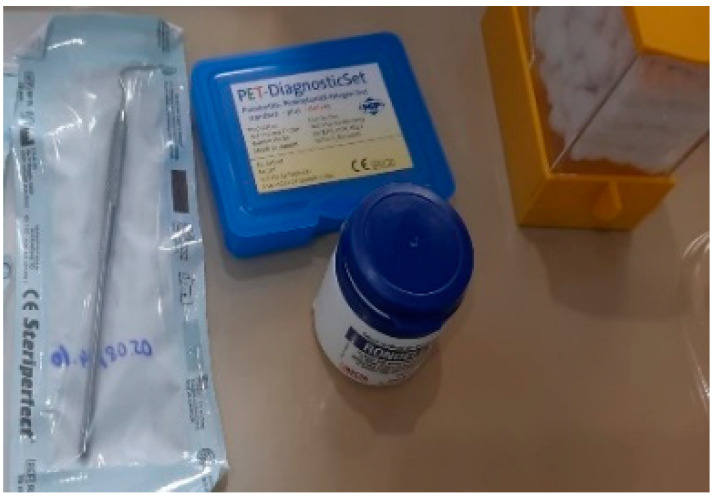
Materials and tools needed for clinical examination.

**Figure 2:**
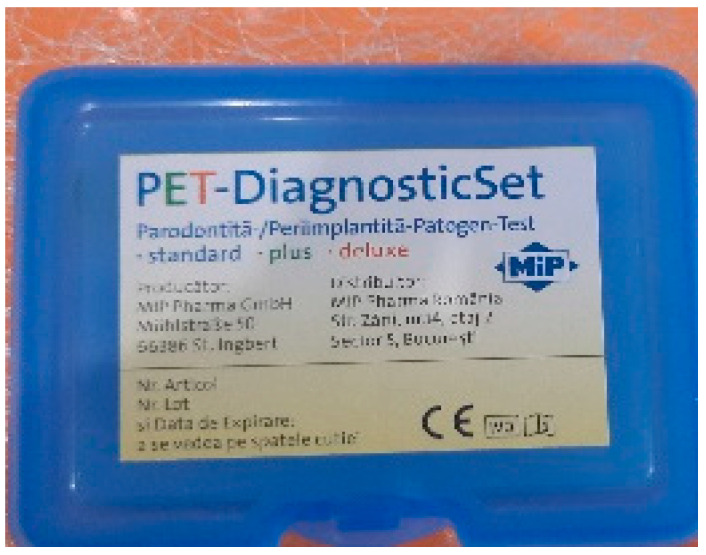
Transport box.

**Figure 3:**
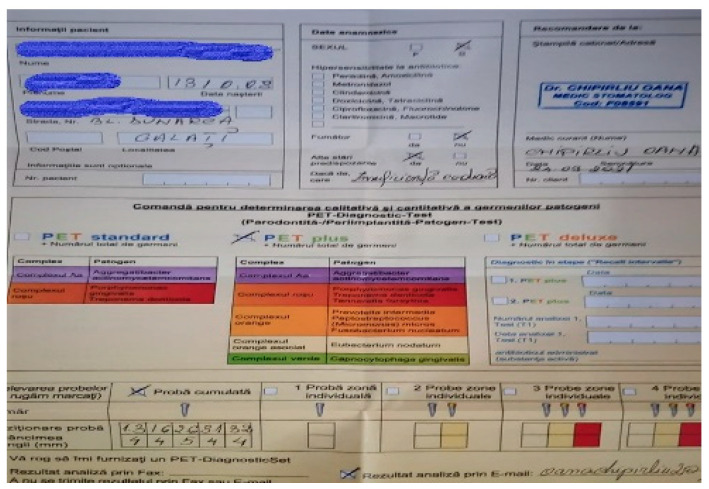
Order form completed with patient data.

**Figure 4:**
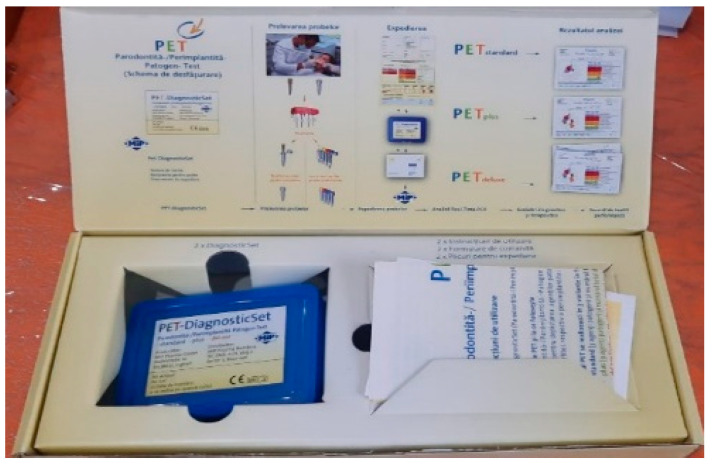
The transport box and the form to fill in for analyses.

**Figure 5:**
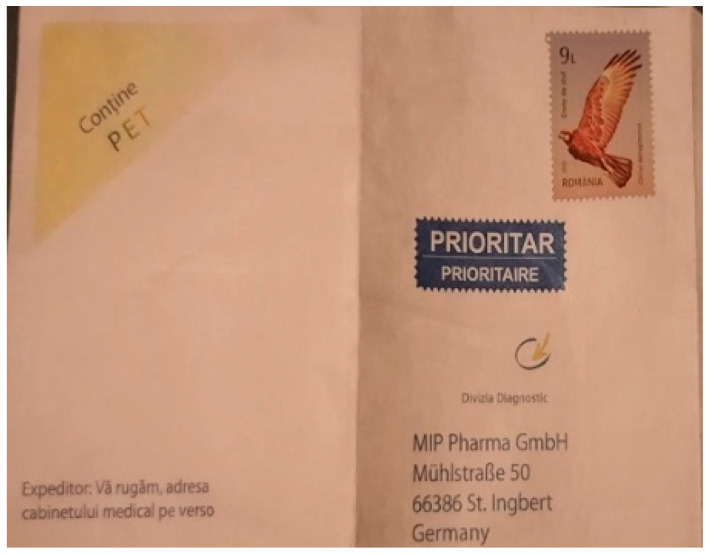
Envelope required for sending the test.

**Figure 6:**
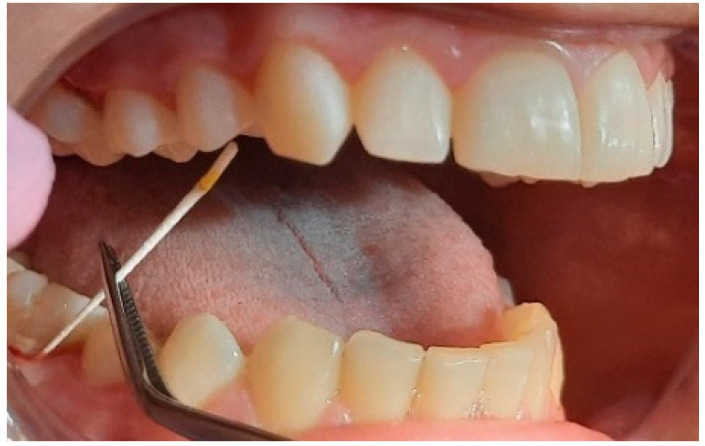
Harvesting at the level of sextant 6, site 4.6. Patient: 14 years old, female, with localized gingivitis, medium, group A.

**Figure 7:**
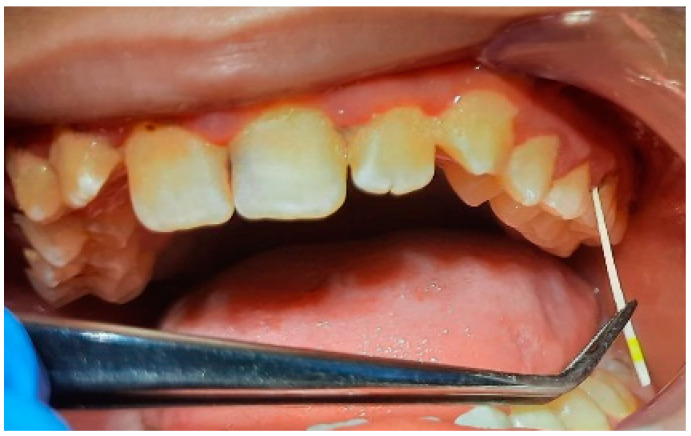
Collection at sextant level 3, site 2.6. Patient: 10 years old with generalized, severe gingivitis, group A.

Microbiological samples were collected, stored, and transported to the laboratory in accordance with the requirements and regulations provided. The microbiological evaluation was carried out by laboratory doctors in the Diagnostic Division of MIP Pharma. The tests were repeated three months after application of the specialized periodontal treatment for both groups (Figure 1, Figure 2, Figure 3, Figure 4, Figure 5, Figure 6 and Figure 7).

### 2.5. Specialist Treatment

Patients received professional hygiene (professional brushing, scaling, and airflow) and procedures applied according to clinical needs, namely topical applications of antiseptic substances, instruction for proper home hygiene, and the recommendation of mouthwash with chlorhexidine 0.12% for 14 days. Patients were reassessed three months after treatment.

Regarding the treatments and manipulations performed, the patients were subjected to a periodontal treatment protocol (initial stage) which consisted of motivation to maintain periodontal health through rigorous oral hygiene instruction (learning the correct brushing techniques and use of oral care aids), supragingival descaling (ultrasonic or manual where needed), airflow (where appropriate), and professional brushing to create favorable local conditions for healing and maintaining good gingival health. The recommendation of mouthwashes with chlorhexidine 0.12% for three weeks was made for severe and medium forms of gingival inflammation via local applications in the office with antiseptic substances (chlorhexidine (0.20%), gel with chlorhexidine (1%), glycosite gel, and gel with metronidazole). For children aged between 7 and 12 years, professional brushing, carried out in the office, was sufficient to remove the microbial factor from the oral cavity.

**Figure 8:**
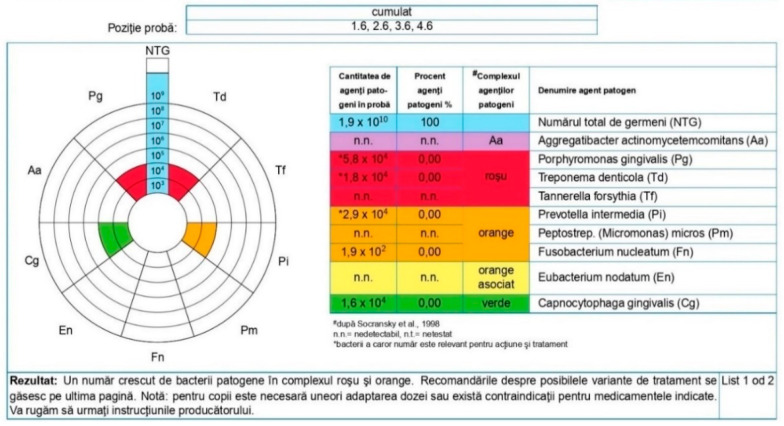
Group A patient. Generalized biofilm-induced gingivitis in medium form. Aged 14 years, female sex, essential hypertension, microbiological results, and initial consultation.

**Figure 9:**
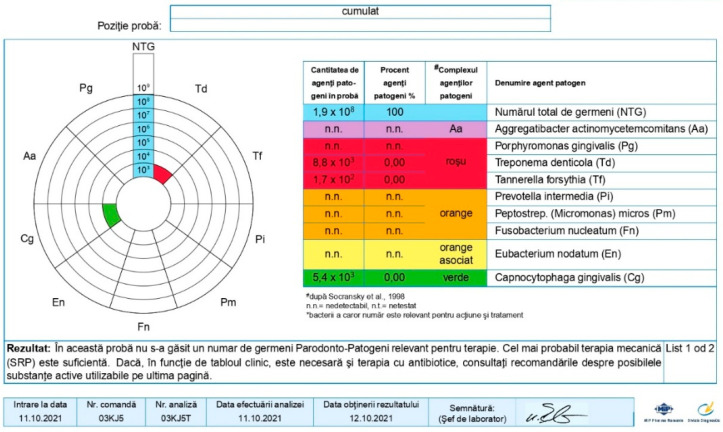
Group A patient’s periodontal health. Aged 14 years, female sex, essential hypertension, and microbiological results 3 months after treatment.

**Figure 10:**
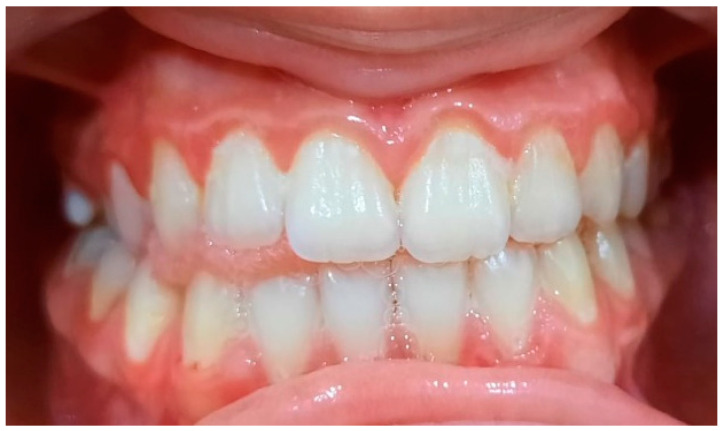
Biofilm-induced gingivitis, medium form, generalized. Female, 14 years old, group A.

**Figure 11:**
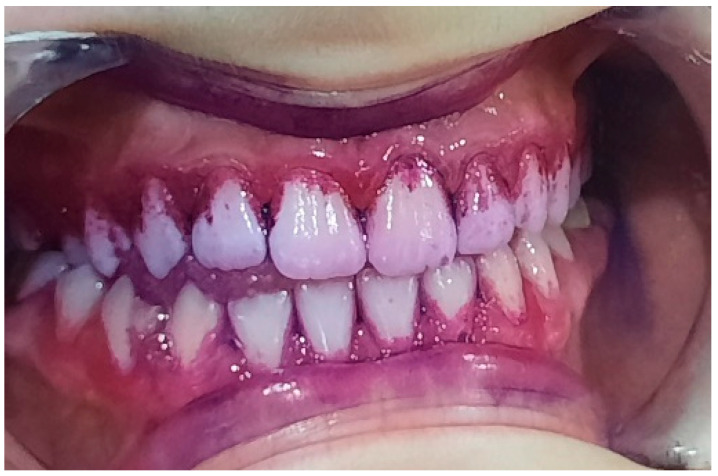
Bacterial plaque staining. IP = 72%, GI = 2. Poor oral hygiene.

**Figure 12:**
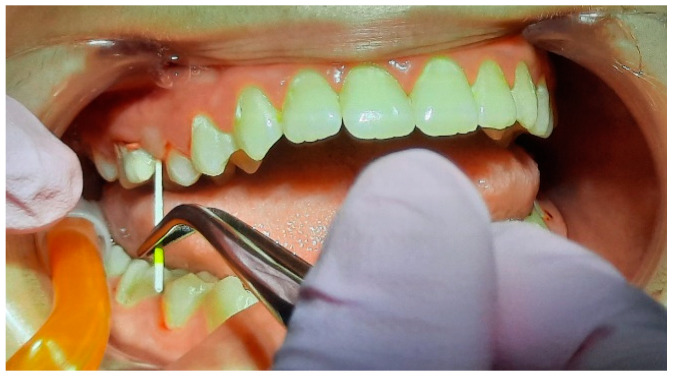
Collection of samples for PET tests (1.6).

**Figure 13:**
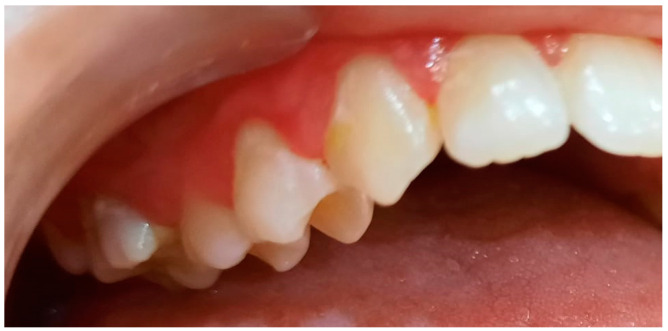
Inflammation of interdental papillae, dental malpositions, and bacterial plaque deposits.

**Figure 14:**
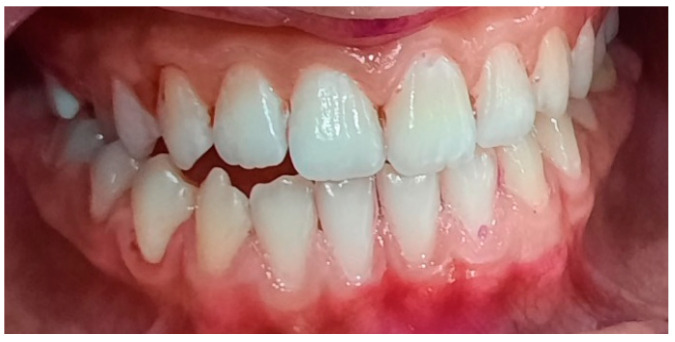
Appearance after professional sanitization.

**Figure 15:**
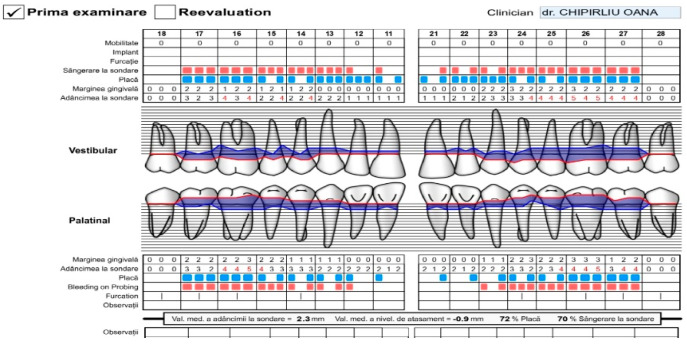
Periodontal record of patient with biofilm-induced gingivitis, medium form, generalized. Female, 14 years, group C. Initial consultation IP = 72%, ISG = 72%, maxilla. (the points in blue are the presence of bacterial plaque on the tooth, the points in red are the bleeding on the probe).

**Figure 16:**
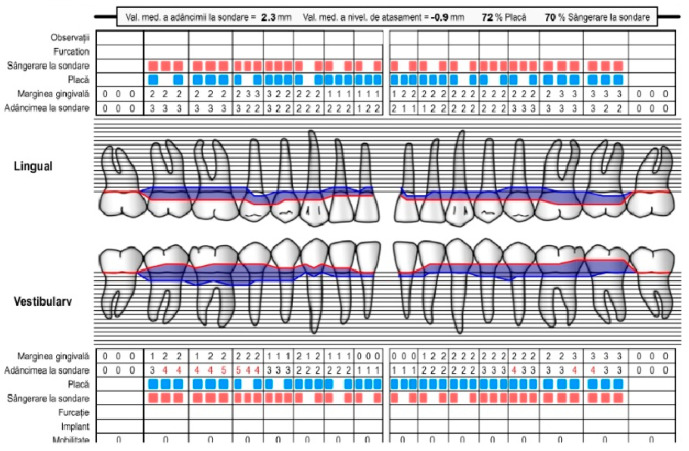
Periodontal record of patient with biofilm-induced gingivitis, medium form, generalized. Female, 14 years, group C. Initial consultation IP = 72%, ISG = 72%, mandible. (the points in blue are the presence of bacterial plaque on the tooth, the points in red are the bleeding on the probe).

**Figure 17:**
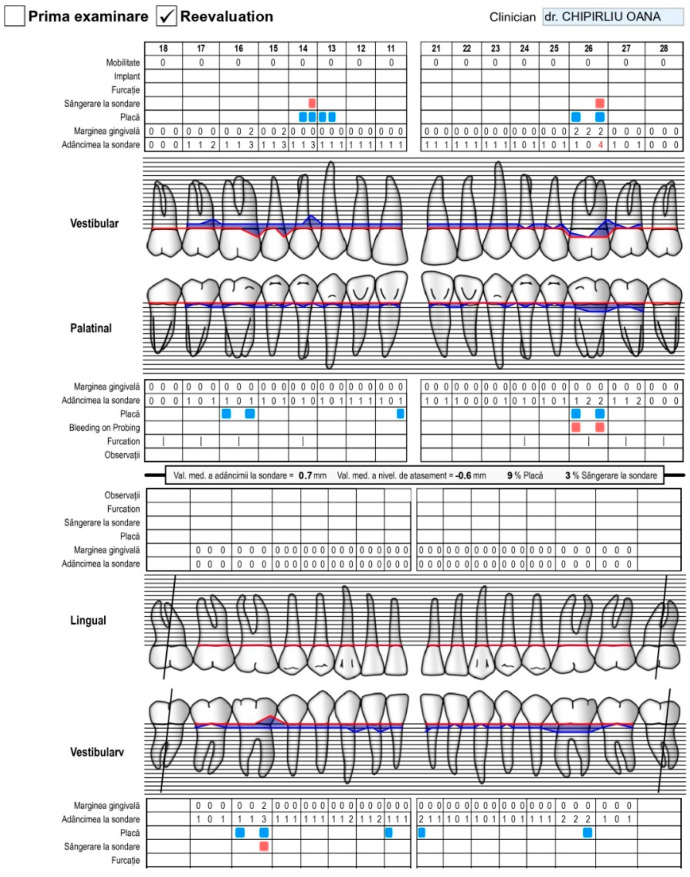
Periodontal patient record. Diagnosis of periodontal disease. Female, 14 years old, group C. Consultation after 3 months. IP = 9%, IS = 3%. Favorable response to the applied treatment and much better hygiene (maxilla and mandible) (the points in blue are the presence of bacterial plaque on the tooth, the points in red are the bleeding on the probe).

The evaluation of the effectiveness of the treatment performed in the local periodontal conditions and the assessment of the effectiveness of the applied therapy were carried out by recording the values of the hygiene and inflammation indices and with microbiological tests, which were repeated three months after the specialized treatment. A bacterial plaque developer was used to reveal the bacterial plaque, which differently colored the bacterial plaque older than three days in dark blue and the newer bacterial plaque in pink (Figure 8, Figure 9, Figure 10, Figure 11, Figure 12, Figure 13, Figure 14, Figure 15, Figure 16 and Figure 17).

### 2.6. Statistical Data Analysis

The data obtained were centralized in a spreadsheet in Microsoft Excel 2011. Microbial species values were edited from text format to scientific format, with exponential notation. To compare the data, statistical tests (Welch’s *t*-test) were used to verify whether or not there were significant differences between the values of the indicators (NTG, Aa, En, etc.) at the initial moment (T1) versus the final one (T2), for the two lots (considered A + C together and separately as A and C). The mean values, standard deviations, and various statistical information (t-statistics, degrees of freedom (df), *p* value, and significance) for the two moments in each separate comparison are presented in tables and, visually, in graphs. Descriptive statistical analyses were performed for the analyzed characteristics, with the results obtained being expressed as the maximum, minimum, and average values and standard deviations for the numerical variables and as a frequency for the categorical variables. For each of the two groups of patients taken into the study, as can be seen, there are several sets of graphs. The last three for each report are boxplots made in the R programming language and include the information related to the statistical test performed at the top. This information can also be found in the tables. The other graphs were generated in Excel, and they are also boxplots like those in made with R.

## 3. Results

All 62 patients were entered into a follow-up program with a visit at baseline and 3 months after treatment. This chapter was structured with four analyses: analysis of epidemiological characteristics; statistical analysis of changes in clinical parameters and diagnosis of periodontal damage at T1 and T2 (at the time of the initial consultation and three months after treatment, respectively); statistical analysis of the predominance of Gram-negative germs in the periodontal inflammation areas at T1 but also after specialized treatment at T2; and analysis of the correlation between clinical (periodontal) parameters and PCR determinations as well as their changes in each batch.

### 3.1. Analysis of Demographic Data

The analysis of the epidemiological characteristics followed the age and gender of the patients in the two groups, and no significant changes were identified between them.

**Figure 18:**
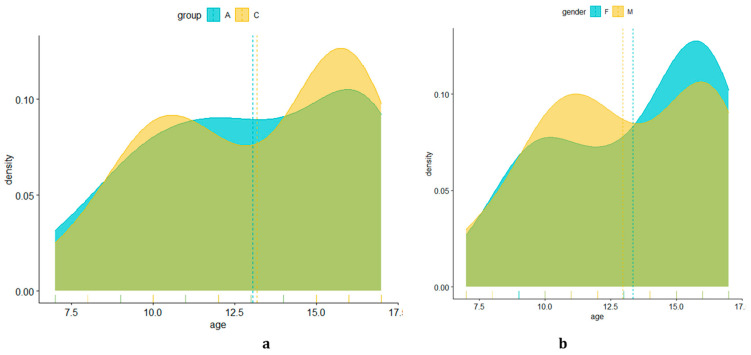
Probability density, estimated based on age of subjects in the two groups (**a**) and by gender (**b**).

**Figure 19:**
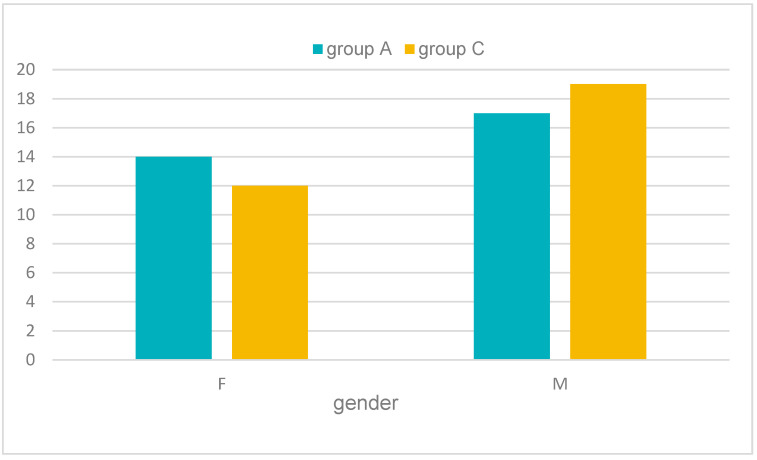
Number of subjects in each batch by gender. Group A = subjects with cardiovascular disease. Group C = subjects without cardiovascular disease.

(1) For the ages of the subjects in the two groups, no significant differences were observed. The average age for group A was 13 years, and for group C, it was 14 years (data presented in Table 1 and Figure 18).

(2) For the genders of the patients in the two groups, a greater presence of males was observed, with 17 patients in group A and 19 patients in group C compared with 14 female patients in group A and 12 female patients in group C (Table 2 and Figure 19).

Statistical analysis of the changes in the clinical parameters and periodontal disease diagnosis at T1 (starting time or initial consultation) and T2 (three months after treatment) was carried out.

### 3.2. Analysis of Clinical Data

**Figure 20:**
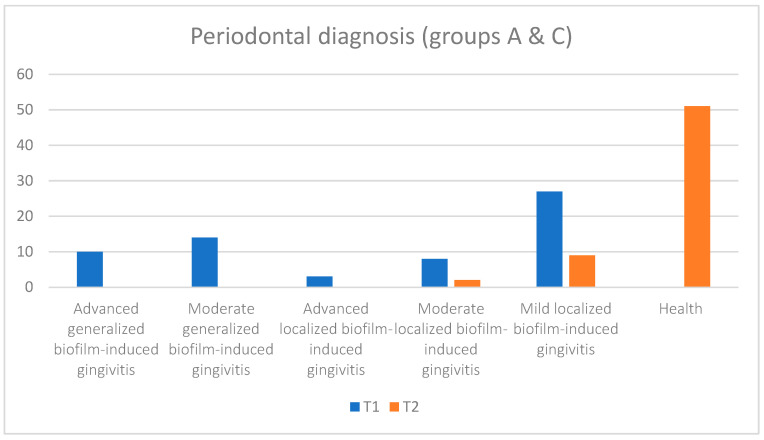
Periodontal diagnosis for subjects from both groups A (subjects with cardiovascular disease) and C (subjects without cardiovascular disease) at initial (T1 = initial consultation) and final (T2 = three months after treatment) times.

**Figure 21:**
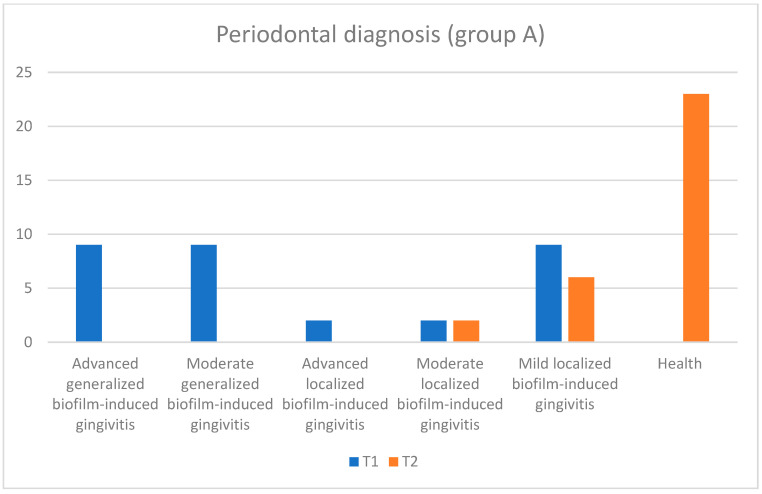
Periodontal diagnosis for subjects in group A (subjects with cardiovascular diseases) at the initial (T1 = initial consultation) and final (T2 = three months after treatment) moments.

**Figure 22:**
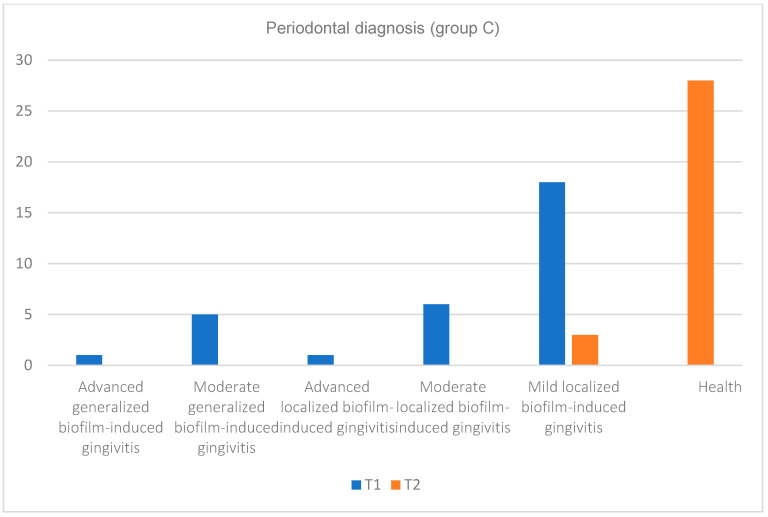
Periodontal diagnosis for subjects in group C (subjects without cardiovascular disease) at the initial (T1 = initial consultation) and final (T2 = three months after treatment) moments.

Regarding the periodontal diagnosis, in Table 3 and Figure 20, Figure 21 and Figure 22, an improvement in health status can be observed at the final moment (T2) after the administration of treatment compared with the initial moment (T1). If, initially, the number of subjects affected by more advanced or medium forms of gingivitis (depending on the severity) (or depending on the extent (generalized or localized)), especially generalized gingivitis, was well represented in both groups, then at the final moment, these forms reduced to zero, and only a few forms of localized gingivitis were encountered.

Also, although at the initial moment, there were subjects with periodontal health at the end in none of the groups, at the time of T2, more than 80% of the subjects were healthy (about 74% in group A and 90% in group C), and the patients who were still affected by periodontal disease suffered from mild (approximately 15%) and moderate (approximately 3%) forms of localized gingivitis. Moreover, in the case of group C, at the final moment, the subjects suffered only from mild localized forms of gingivitis, and their number was reduced (approximately 10%). A better response to the applied treatment was observed in subjects with a good general condition and without cardiovascular disease. We can conclude that most of the eventual tissue destruction was caused indirectly by the host’s response to infection.

The clinical parameters of the IP (oral hygiene) and IG (degree of inflammation) were analyzed at T1 and T2 after specialized treatment. As in the case of periodontal diagnosis, for gingival inflammation, analyzed using the GI, a shift to the right of the histograms of the distribution of subjects according to the gingival index from the initial time (T1) to the final time (T2) can be seen, corresponding to an obvious improvement in health status (see Table 4). There were no subjects with healthy gums and normal appearances initially, but in the end, their number increased substantially to over 80% in both groups, and the number of subjects with advanced inflammation was reduced to zero. Moreover, in the case of the subjects in group C, there were no more cases of moderate or advanced gingival inflammation and only cases of subjects with normal-appearing gums (approximately 90%) and cases with mild inflammation (approximately 10%) (Table 4, Table 5 and Table 6).

Oral hygiene also changed considerably. After specialized treatment, the PI changed from maximum values of 84% to maximum values of 35% at the time of T2 for the total group. For each batch of group A, with maximum values of 84% at T1, it changed to maximum values of 35% at the time of T2. For group C, the value at T1 was 65%, and at T2, it was 31%. The PI values were consistent with the periodontal disease diagnoses, which were more severe in the group of patients with cardiovascular diseases.

**Figure 23:**
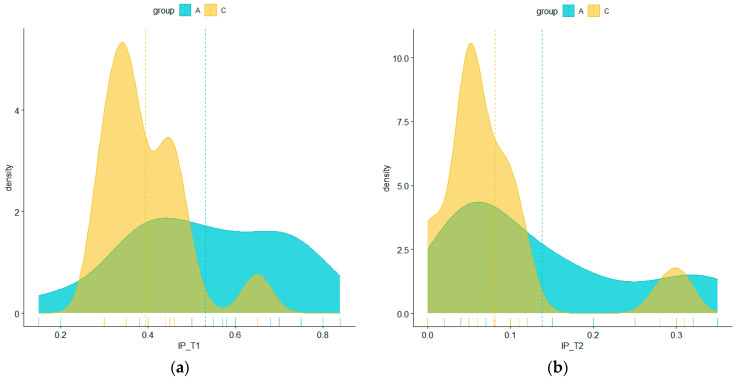
Probability density estimated based on IP values in the two batches at T1 (initial consultation) (**a**) and T2 (three months after treatment) (**b**).

Table 5 and Table 6 present the distribution of the main statistics (minimum, maximum, quartile, median, mean, and outliers) for the values of the IP parameter in the case of the two batches, both at the initial time and at the end time. A significant decrease in values between the two moments is visible. The statistical analysis is summarized in Table 5 and Table 6 and represented graphically in Figure 23a,b.

With the help of Welch’s *t*-test, the mean IP values for groups A and C were compared. From here, it can be seen that the differences between the mean IP values at the two moments, T1 and T2, were statistically significant regardless of the group taken into account, both when they were considered together and when they were analyzed separately.

Also, as can be seen in Figure 23a,b, the values of the IP parameter in the case of the two lots showed a similar evolution, with a shift to the left (toward lower values for the parameter) and the shape of the probability density function undergoing changes through a displacement and agglomeration of values in the same decreasing direction.

### 3.3. Results of Microbiological Determinations

**Figure 24:**
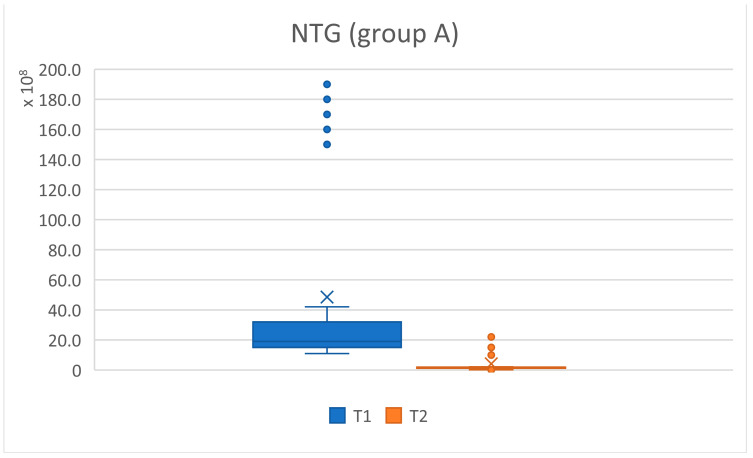
NTG values for patient group A (subjects with cardiovascular diseases) at initial (T1 = initial consultation) and final (T2 = three months after treatment) times.

**Figure 25:**
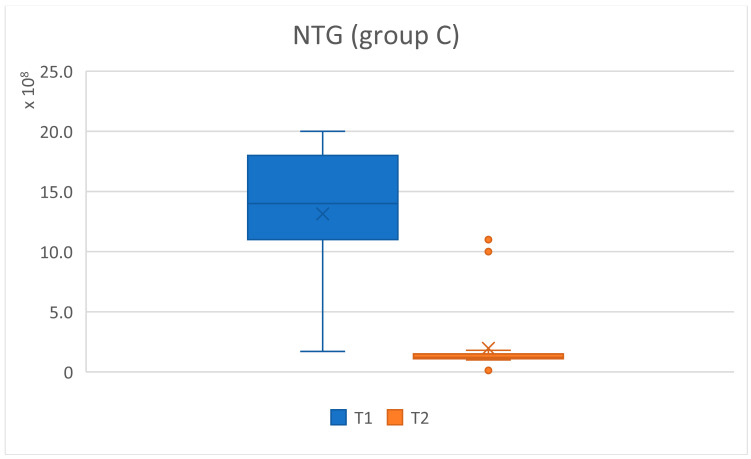
NTG values for patient group C (subjects without cardiovascular disease) at initial (T1 = initial consultation) and final (T2 = three months after treatment) times.

**Figure 26:**
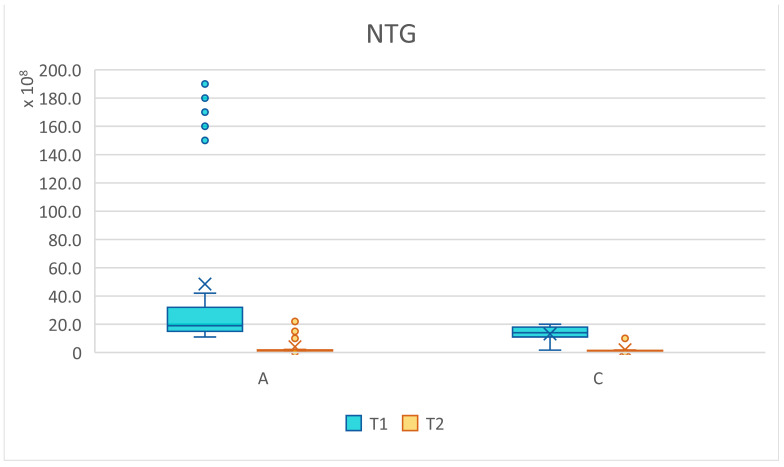
NTG values for groups A (subjects with cardiovascular disease) and C (subjects without cardiovascular disease) at the initial (T1 = initial consultation) and final (T2 = three months after treatment) times.

The total number of germs (NTG) present in a sample at baseline and three months after treatment was compared. A statistically significant difference was observed, with a representative reduction in the total number of germs which was higher in the patients with cardiovascular diseases. Using Welch’s *t*-test, we examined whether the means of the analyzed batches differed significantly, with a level of significance given by *p* values < 0.05, and *p* < 0.001 was considered highly statistically significant for the values compared. The null hypothesis considered that there were no differences between the two groups (Table 7 and Figure 24, Figure 25 and Figure 26).
**Signif. Codes*****p* Value****Interpretation******aproximately 0Extremely strong evidence against the null hypothesis***0 < *p* < 0.001Rather strong evidence against the null hypothesis**0.001 ≤ *p* < 0.01Strong evidence against the null hypothesis*0.01 ≤ *p* < 0.05Moderate evidence against the null hypothesis.0.05 ≤ *p* < 0.1Weak evidence against the null hypothesisns0.1 ≤ *p*Insignificant, with a lack of evidence against the null hypothesis (the data are consistent with the null hypothesis) 

After analyzing the PET tests, the microbiological test values for nine periodontopathogens were observed: *Aggregatibacter actinomycetemcomitans* (Aa), *Porphyromonas gingivalis* (Pg), *Treponema denticola* (Td), *Tannerella forsythia* (Tf), *Prevotella intermedia* (Pi), *Peptostrep.* (*Micromonas*) *micros* (Pm), *Fusobacterium nucleatum* (Fn), and *Capnocytophaga gingivalis* (Cg).

For the total number of germs present in the analyzed samples, a significant decrease was observed from T1 to T2 after applying the treatment, which proves that the alternative hypothesis is true. There were differences between the average values of the microbial load from T1 to T2. More statistically representative changes occurred at the level of batch C, marked with four stars (****).

The graphic representation is also significant and shows the changes that occurred in the NTG for batches A and C, regarding the oscillations of the average values. For group A (study), the average at T1 NTG = 4.85 × 10⁹, and at T2, NTG = 4.13 × 10⁸. For group C, at T1 (control) NTG = 1.31 × 10⁹, and at T2, NTG = 1.96 × 10⁸ (Figure 24, Figure 25 and Figure 26).

**Figure 27:**
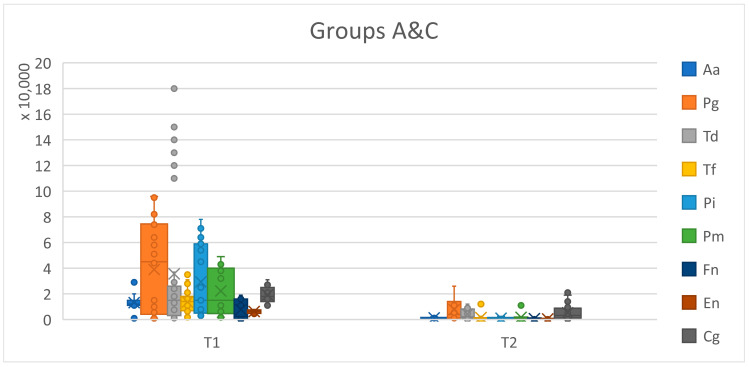
Graphical representation showing the presence of periodontal pathogens at T1 and T2 (T1 = initial consultation; T2 = three months after treatment).

The values of the microbial species before and after treatment were evaluated, and at the level of the total batch, significant changes were observed. At time T1, *Aggregatibacter actinomycetemcomitans* (Aa) was detected in 11 (17.7%) patients and only in 5 (8.1%) patients, at time T2, *Porphyromonas gingivalis* (Pg) was detected at T1 in 45 (72.6%) patients and 18 (29.0%) at T2, *Treponema denticola* (Td) was present in 46 (74.2%) patients at T1 and in 36 (58.1%) patients at T2, *Tannerella forsythia* (Tf) was found in 52 (83.9%) patients at T1 and 32 (51.6%) at T2, *Prevotella intermedia* (Pi) was present in 37 (59.7%) patients at T1 and 25 (40.3%) after applying the treatment, *Peptostrep.* (*Micromonas*) *micros* (Pm) was found in 21 (33.9%) patients at T1 and 16 (25.8) at T2, *Fusobacterium nucleatum* (Fn) was detected in 49 (49.0%) patients at T1 and 17 (27.4%) at T2, and *Capnocytophaga gingivalis* (Cg) was found in 62 (100%) patients, being encountered in the state of periodontal damage and compatible with periodontal health. The main changes in the values are shown in Table 8 and Figure 27, where we observe a large representation of microorganisms from the red complex for both batches before treatment and a significant reduction in them after the specialized treatment. The presence of statistically significant differences, marked with three and four stars, is noted.

Statistical analysis was performed only for the detected values. Undetected values meant rather small values, falling below a specific threshold value for laboratory analysis, which is represented by values lower than 1 × 10^2^ (values noted with n.n (undetectable)).

Concomitant with the decrease in the average of the detected values, an increase in the number of undetectable values at the final moment was observed, which can be concluded to be an increase in the number of subjects with a good periodontal health status.

The changes were significant for the values of all types of microorganisms, with the exception of *Capnocytophaga gingivalis*, whose values remained constant in both the affected state and after treatment, also being compatible with the periodontal health state. *Porphyromonas gingiv*, *Treponema denticola*, *Tannerella forsythia*, and *Fusobacterium nucleatum* (Fn) were the most common microbial species in periodontal disease. *Fusobacterium nucleatum* (Fn), found in 25% of group A and 24% of group C, was considerably reduced after the specialized treatment, being responsible for the adhesion of other pathogens. *Treponema denticola* and *Tannerella forsythia* represented the most numerous species from the red complex. At the level of the whole lot, *Treponema denticola* was present at a cumulative rate of 74.2% at T1 (41.9% in group A and 32.3% in group C). As for *Tannerella forsythia*, it was present in 83.9% of the total lot, of which 41.9% were in group A and 41.9% were in group C (Table 9).

## 4. Discussion

In our study, we examined the prevalence of nine species of periodontopathogenic, Gram-negative, anaerobic microorganisms, and all of these nine species were found in the patients in the study group as well as in the control group.

We also analyzed the changes in clinical parameters through the state of hygiene (PI) and inflammation (IG), which were recorded at T1 and T2 (3 months after treatment), being analyzed in the context of the group with cardiovascular disease compared with the witness group.

During the last few decades, there has been a growing interest in the impact of oral health on atherosclerosis and cardiovascular disease, especially regarding the early detection of these connections.

The infection–inflammation–atherosclerosis–cardiovascular disease–periodontal disease relationship has been described and supported in vast studies in the medical literature. Periodontal disease presents a quite varied spectrum of oral manifestations from gingivitis to periodontitis, with both presenting mild, moderate, severe, localized, or generalized forms that lead to alveolar bone loss and tooth loss.

*Aggregatibacter actinomycetemcomitans* (Aa) was present in high concentrations but relatively low numbers in the bacterial plaque of the group with cardiac pathology and much lower numbers in the control group. The low prevalence of Aa (17.7%) at the level of the total group (8.1% in group A and 6.5% in group C) before treatment confirmed its association with severe forms of deep periodontal damage, which are forms rarely found in children and adolescents.

In the case of children with cardiovascular diseases (included in group A), the forms of generalized gingivitis were more numerous compared with the control group (group C), and the response to treatment was not as good. In group A, there were nine patients with severe generalized gingivitis, and in group C, only one patient had this diagnosis. In group C, there were 18 patients with mild and localized forms of gingivitis, and in group A, there were nine patients with this diagnosis. After the specialized treatment, in group A, 74.2% of the patients received the periodontal health diagnosis, and in group C, a significant proportion of 90.3% of the patients were presented with this diagnosis.

The data obtained by our study are consistent with previous research. Nosrati Erez et al. published a study in 2013 which was carried out on 50 children, with 25 in the study group, which was represented by children with cardiovascular and congenital conditions, and 25 children who did not present these conditions, representing the control group. The ages of the children were between 7 and 13 years. The children in the study group presented more severe gingival diseases and plaque and tartar deposits and a worse response to the applied treatment compared with the control group [26].

The mean PI values for group A were 50% at T1 and 10% at T2, consistent with other studies in both adults and children or youths. In a study carried out by Shetty et al. in 2012 in India with 200 subjects of different age groups, the patients with cardiovascular disease had worse oral hygiene conditions compared with the control group of healthy patients, and the condition of periodontal inflammation was more common in the study group [26,27].

Another randomized cohort study published in 2022 by Kim et al., was conducted with 51,677 patients with HTN, who were evaluated by the Oral Health View between 2003 and 2004. They were followed up with in 2015, a duration of 11 years, and the following were observed: inadequate oral hygiene and the presence of the risk of developing a vascular accident in the case of patients with HTN [32].

Microorganisms from the red complex, such as *Porphyromonas gingiv* (Pg), *Treponema denticola* (Td), and *Tannerella forsythia* (Tf), were identified in quite a large number among patients with cardiovascular disease (Pg = 40.3%, Td = 41.9%, and Tf = 41.9% of patients in group A). Previous studies have highlighted the fact that these microorganisms are the most common ones in atheroma plaque [29,33]. The presence of periodontal infection can lead to episodes of bacteremia, with the inoculation of arterial atherosclerotic plaques with periodontal pathogens such as *Porthyromonas gingivalis*, *Actinobacillus actinomycetemcomitans*, *Bacteroides forsythus*, and *Treponema denticola* which, through development and multiplication, increase the inflammatory process, a condition that leads to atherosclerotic plaque instability.

*Porphyromonas gingival* (Pg) was identified in a large number of patients from group A and also those from group C (40.3% and 32.3%, respectively), a fact also confirmed by previous studies [34,35,36,37,38].

From the orange complex, *Fusobacterium nucleatum* (Fn) was the most common microorganism in the two groups before the specialized treatment. Other studies have associated the presence of Fn and Pg with the early stages of periodontal damage.

*Fusobacterium nucleatum* (Fn) possesses numerous virulence factors, including adhesins, which facilitate adhesion to and invasion of various cell types, leading to not just colonization, dissemination, and triggering the host’s immune response but also endotoxins and serine protease secretion, which are responsible for suppressing the nutritional needs of other microorganisms [36,39].

The yellow and green complexes represented by *Eubacterium nodatum* (En) and *Capnocytophaga Gingivalis* (Cg), respectively, are complexes with different actions. *Eubacterium nodatum* (En) was found in a small number of patients (five in batch A (8.1%) and three in batch C (3.2%)), showing results consistent with previous studies carried out by Debar Hell et al. in 2012 on 76 patients with aggressive periodontitis and 185 patients with chronic periodontitis, where En was detected in patients with aggressive forms and Cg was found in patients with chronic forms [33,39,40].

Microorganisms from the green complex, represented by *Capnocytophaga Gingivalis* (Cg), which are also compatible with the state of periodontal health and represent only a substrate for the other microorganisms, were found in equal numbers in the states of periodontal damage and periodontal health, being the most common microorganism. These results are consistent with previous studies [11,40].

## 5. Conclusions

In our study, we wanted to highlight the existing association between the subgingival microbiota and local clinical signs of periodontal damage. We also followed the response to specialized treatment in patients with cardiovascular disease and analyzed how these diseases influence the patients’ response to treatment.

The composition of the subgingival microbial plaque was directly influenced by the degree of oral hygiene, but the response to specialized treatment was influenced by the general health status.

The results of this study support the conclusion that periodontal pathogens appear and multiply in the absence of proper hygiene during childhood after the eruption of permanent teeth, and their action leads to the initiation of periodontal disease.

In children with cardiovascular disease and gingival inflammation, the association of 5–7 microbial species was identified, depending on the type (localized or generalized with medium or severe gingivitis). For the subjects without cardiovascular disease, the association of 4–7 microbial species was observed before treatment. For both groups, the microbial species decreased significantly after treatment, with more visible results for group C.

In light of the current evidence, inflammatory diseases of the oral cavity can increase the severity of cardiovascular disease.

Considering the global burden of periodontal diseases, it is important to develop meaningful therapeutic directions for the prophylaxis of periodontal diseases and contamination with periodontopathogens.

If periodontitis is one of the major causes of tooth loss in adulthood, then through the quality of periodontal treatments and specialist consultation, even from childhood, this phenomenon can be prevented.

## Figures and Tables

**Table 1:** Summary of main statistical values for age of subjects.

		Age					
Groups	N	Min	1st Qu.	Median	Mean	3rd Qu.	Max
all	62	7	10	13.5	13.13	16	17
group A	31	7	10.5	13	13.06	16	17
group C	31	7	10	14	13.19	16	17

**Table 2:** Genders of subjects in each group.

		Gender			
Groups	N	F		M	
all	62	26	41.94%	36	58.06%
group A	31	14	45.16%	17	54.84%
group C	31	12	38.71%	19	61.29%

**Table 3:** Periodontal diagnosis at the initial (T1 = initial consultation) and final (T2 = three months after treatment) times.

		Periodontal Diagnosis											
		Advanced Generalized Biofilm-Induced Gingivitis		Moderate Generalized Biofilm-Induced Gingivitis		Advanced Localized Biofilm-Induced Gingivitis		Moderate Localized Biofilm-Induced Gingivitis		Mild Localized Biofilm-Induced Gingivitis		Health	
Groups	N	T1	T2	T1	T2	T1	T2	T1	T2	T1	T2	T1	T2
all	62	10	0	14	0	3	0	8	2	27	9	0	51
group A	31	9	0	9	0	2	0	2	2	9	6	0	23
group C	31	1	0	5	0	1	0	6	0	18	3	0	28

**Table 4:** Gingival index values per group.

		Gingival Index							
		Gums with Normal Appearance (Cod 0)		Gums with Mild Inflammation (Cod 1)		Gums with Moderate Inflammation (Cod 2)		Gums with Advanced Inflammation (Cod 3)	
Groups	N	T1	T2	T1	T2	T1	T2	T1	T2
all	62	0	51	26	9	23	2	13	0
group A	31	0	23	9	6	11	2	11	0
group C	31	0	28	17	3	12	0	2	0

**Table 5:** Summary of the main statistical values for the IP parameter.

		IP											
		Min		1st Qu.		Median		Mean		3rd Qu.		Max	
Groups	N	T1	T2	T1	T2	T1	T2	T1	T2	T1	T2	T1	T2
all	62	15.00%	0.00%	35.00%	5.00%	42.00%	6.50%	46.21%	10.94%	56.50%	14.25%	84.00%	35.00%
group A	31	15.00%	2.00%	40.00%	5.00%	50.00%	10.00%	53.06%	13.77%	70.00%	20.00%	84.00%	35.00%
group C	31	30.00%	0.00%	35.00%	5.00%	35.00%	5.00%	39.35%	8.10%	45.00%	10.00%	65.00%	31.00%

**Table 6:** Results of Welch’s t statistical test, with comparisons between initial (T1) and final (T2) moments.

		IP							
		T1		T2		t	df	*p* Value	Signif
Groups	N	Mean	±SD	Mean	±SD				
all	62	46.21%	15.67%	10.94%	9.94%	14.97	102.28	1.28 × 10^-27^	****
group A	31	53.06%	17.84%	13.77%	11.06%	10.42	50.09	3.83 × 10^-14^	****
group C	31	39.35%	9.16%	8.10%	7.88%	14.40	58.71	6.28 × 10^-21^	****

**Table 7:** Results of Welch’s t statistical test, showing comparisons between mean NTG values at initial (T1 = initial consultation) and final (T2 = three months after treatment) time points, **** Extremely strong evidence against the null hypothesis, *** Rather strong evidence against the null hypothesis.

		NTG							
		T1		T2		t	df	*p* Value	Signif
Groups	N	Mean	±SD	Mean	±SD				
all	62	3.08 × 10^9^	4.66 × 10^9^	3.05 × 10^8^	4.90 × 10^8^	4.66	62.35	0.0000169	****
group A	31	4.85 × 10^9^	6.12 × 10^9^	4.13 × 10^8^	6.20 × 10^8^	4.02	30.62	0.000354	***
group C	31	1.31 × 10^9^	5.86 × 10^8^	1.96 × 10^8^	2.83 × 10^8^	9.56	43.24	3.16 × 10^-12^	****

**Table 8:** Results of Welch’s statistical *t*-test, showing comparisons between the prevalence of periodontal pathogens at the initial (T1) and final (T2) time points for groups A and C (A = subjects with cardiovascular disease; C = subjects without cardiovascular disease) (*** Rather strong evidence against the null hypothesis, **** Extremely strong evidence against the null hypothesis).

			T1			T2		t	df	*p* Value	Signif
Indicator	N	#n.n.	Mean/Val	±SD	#n.n.	Mean	±SD				
Aa	62	51/11Aa	13,181.82	6896.64	57/5Aa	1320	438.18	5.68	10.62	0.000191	***
Pg	62	17/45 Pg	38,984.44	34,805.29	44/18Pg	7722.22	7493.81	5.70	52.95	5.35 × 10^-7^	****
Td	62	16/46 Td	35,613.04	50,352.79	26/36Td	4206.94	3819.53	4.21	45.66	0.000117	***
Tf	62	10/52 Tf	13,707.69	7989.23	32/32Tf	1529.33	2093.62	10.39	62.31	3.13 × 10^-15^	****
Pi	62	25/37 Pi	29,364.86	27,264.38	37/25Pi	1032.40	724.93	6.32	36.08	2.60 × 10^-7^	****
Pm	62	41/21 Pm	22,266.67	17,722.06	46/16	1711.25	2588.38	5.24	21.11	3.33 × 10^-5^	****
Fn	62	13/49 Fn	7757.14	7825.23	45/17	719.41	586.28	6.25	49.53	9.41 × 10^-8^	****
En	62	54/8 En	6212.50	1389.18	57/5En	410	610.04	10.33	10.27	9.47 × 10^-7^	****
Cg	62	0/62 Cg	19,258.06	6316.61	0/62Cg	5285.32	5227.73	13.42	117.88	7.30 × 10^-24^	****

**Table 9:** The prevalence of the 9 bacterial species among the patients included in the study, cumulatively and separately.

Parameter	N (%) Patients Total Lot T1	N (%) Patients Total Lot T2	N (%) Patients Lot A T1	N (%) Patients Lot A T2	N (%) Patients Lot C T1	N (%) Patients Lot C T2
Aa	11 (17.7%)	5 (8.1%)	7 (11.3%)	4 (6.5%)	4 (6.5%)	1 (1.6)
2. Pg	45 (72.6%)	18 (29.0)	25 (40.3%)	10 (16.1)	20 (32.3%)	8 (12.9%)
3. Td	46 (74.2%)	36 (58.1%)	26 (41.9)	19 (30.6)	20 (32.3%)	19 (30.6)
4. Tf	52 (83.9%)	32 (51.6%)	26 (41.9)	16 (25.8%)	26 (41.9%)	16 (25.8%)
5. Pi	37 (59.7%)	25 (40.3%)	20 (32.3%)	13 (21.0%)	17 (27.4%)	12 (19.4%)
6. Pm	21 (33.9)	16 (25.8%)	12 (19.4%)	9 (14.5%)	9 (14.5%)	7 (11.3%)
7. Fn	49 (79%)	17 (27.4%)	25 (40.3%)	9 (14.5%)	24 (38.7%)	8 (12.9%)
8. Enc	8 (12.9%)	5 (8.1%)	5 (8.1%)	3 (4.8%)	3 (4.8%)	2 (3.2%)
9. Cg	62 (100%)	62 (100%)	31 (50%)	31 (50%)	31 (50%)	31 (50%)

## Data Availability

The data presented in this study are available upon request from the corresponding author. The data are not publicly available due to privacy reasons.
